# Deubiquitinase USP19 extends the residual enzymatic activity of phenylalanine hydroxylase variants

**DOI:** 10.1038/s41598-022-18656-0

**Published:** 2022-08-20

**Authors:** Neha Sarodaya, Apoorvi Tyagi, Hyun-Jin Kim, Ju-Seop Kang, Vijai Singh, Seok-Ho Hong, Woo Jin Kim, Kye-Seong Kim, Suresh Ramakrishna

**Affiliations:** 1 Graduate School of Biomedical Science and Engineering, Hanyang University, Seoul, South Korea; 2 Department of Pharmacology, College of Medicine, Hanyang University, Seoul, South Korea; 3 Department of Biosciences, School of Science, Indrashil University, Rajpur, Mehsana, Gujarat India; 4 Department of Internal Medicine, School of Medicine, Kangwon National University, Chuncheon, South Korea; 5 Department of Internal Medicine and Environmental Health Center, Kangwon National University Hospital, Kangwon National University School of Medicine, Chuncheon, South Korea; 6 College of Medicine, Hanyang University, Seoul, South Korea

**Keywords:** Biochemistry, Molecular biology

## Abstract

Phenylalanine hydroxylase (PAH) is a key enzyme in mammals that maintains the phenylalanine (Phe) concentration at an appropriate physiological level. Some genetic mutations in the PAH gene lead to destabilization of the PAH enzyme, leading to phenylketonuria (PKU). Destabilized PAH variants can have a certain amount of residual enzymatic activity that is sufficient for metabolism of Phe. However, accelerated degradation of those variants can lead to insufficient amounts of cellular PAH protein. The optimal protein level of PAH in cells is regulated by a balancing act between E3 ligases and deubiquitinating enzymes (DUBs). In this work, we analyzed the protein expression and stability of two PKU-linked PAH protein variants, R241C and R243Q, prevalent in the Asian population. We found that the tested PAH variants were highly ubiquitinated and thus targeted for rapid protein degradation. We demonstrated that USP19, a DUB that interacts with both PAH variants, plays a regulatory role by extending their half-lives. The deubiquitinating activity of USP19 prevents protein degradation and increases the abundance of both PAH protein variants. Thus, our study reveals a novel mechanism by which deubiquitinating activity of USP19 extends the residual enzymatic activity of PAH variants.

## Introduction

Phenylketonuria (PKU) is the most prevalent inherited metabolic disorder and is characterized by mutations in the phenylalanine hydroxylase (*PAH*) gene that encodes the PAH enzyme (EC1.14.16.1). PAH is crucial for catalyzing L-phenylalanine (Phe) to L-tyrosine (Tyr) in hepatocytes in the presence of cofactor (6R)-5,6,7,8-tetrahydrobiopterin (BH_4_) and molecular oxygen^1,2^. Loss-of-function mutations in the *PAH* gene cause impairment of that rate-limiting step, leading to a toxic accumulation of Phe (> 120 µmol/L) in the blood and consequent developmental disabilities, degenerative neuropathy, and intellectual deficits. In some cases, impaired BH_4_ metabolism can also lead to an elevated Phe concentration in the blood^3–5^. The current clinically approved treatment focuses on a low-Phe diet regime and oral supplementation with BH_4_ (sapropterin, commercially available as Kuvan®)^6^. However, those treatments can be ineffective against PKU due to difficulty in adhering to a low-Phe diet and the inadequate Phe-lowering effect of synthetic supplements. In clinical trials, the combination of sapropterin and dietary Phe restriction was effective in only 20–56% of patients^7,8^. Therefore, it is important to explore novel treatment approaches that can lower the elevated Phe levels in PKU patients.

Most PKU-causing mutations in the *PAH* gene are missense mutations that cause reduced proteolytic stability, aggregation, accelerated thermal unfolding, and rapid degradation^9,10^. The spectrum of PAH mutations has been investigated extensively around the world, and a strong link has been discovered between clinical symptoms and mutational genotypes. Mutations R243Q, IVS4-1G>A, R241C, and E6-96A>G are the most common in Asian PKU patients^11,12^. Despite severe phenotypic alterations, certain PAH missense mutations confer some amount of residual activity, whereas others completely abolish the activity of the enzyme^13,14^. The two most prevalent mutations in Asia, R241C (mild PKU) and R243Q (severe PKU), have been shown to have residual activity of 25% and 12.8%, respectively^11^. Therefore, it is of substantial interest to discover a mechanism, potentially multifactorial, that can protect a PAH variant that has residual activity from rapid protein degradation and extend its protein half-life in vivo.

Post-translational modifications (PTMs), such as acetylation, SUMOylation, nitration, ubiquitination, palmitoylation, glycosylation, phosphorylation, and oxidation, can regulate protein activity, the rate of protein turnover, the accumulation of aggregates, and the degradation of toxic disease-causing proteins^15^. Among all the PTMs, a major regulator of cellular protein turnover is the ubiquitin proteasome system (UPS). The process of UPS-mediated degradation occurs when ubiquitin‐activating enzyme (E1), ubiquitin conjugating-enzyme (E2), and ubiquitin ligase (E3) enzymes modify target proteins with ubiquitin chains. The ubiquitinated proteins are then recognized and degraded by the 26S proteasome. However, deubiquitinating enzymes (DUBs) can rescue the ubiquitinated substrate proteins from degradation by catalyzing the removal of the ubiquitin molecules from the target proteins. Thus, a balance between ubiquitination and deubiquitination is essential to maintain a healthy pool of cellular proteins^16^.

Several reports have proposed mechanisms for the proteolytic degradation of PAH by the ubiquitin-dependent proteasomal degradation pathway^17–19^. We previously identified the specific E3 ligase APC/C^Cdh1^ and deubiquitinase USP19 responsible for modifying PAH wild-type (PAHwt) protein to balance the ubiquitination and deubiquitination of PAH in cells^19,20^. Our data demonstrated that PAHwt undergoes Cdh1-mediated ubiquitination and rapid degradation, which decreases the half-life of the PAHwt protein^19^. In contrast, USP19 reverses the ubiquitination of the PAHwt protein and extends its half-life. In other words, *Cdh1* knockdown and USP19 supplementation increased PAHwt protein expression and thereby its enzymatic activity. Therefore, we decided to investigate whether USP19 has a similar deubiquitinating effect on PAH variants, which could protect them from rapid degradation and improve their metabolic function.

In this study, we show that two PKU disease–associated PAH variants, R241C and R243Q, are expressed at lower levels than PAHwt and are highly ubiquitinated for rapid degradation by the proteasomal pathway. We also demonstrate that USP19 deubiquitinates and rescues both the R241C and R243Q PAH variants from rapid protein degradation, whereas inhibiting USP19, either by sgRNA-mediated knockdown or by broad spectrum DUB inhibitor, promoted the ubiquitination of these two PAH variants. USP19 extends the half-lives of the R241C and R243Q variants thus maintaining sufficient PAH enzyme in the cells. Moreover, the associated increase in PAH protein levels resulted in extended residual activity of both PAH variants in the presence of USP19. Our study provides novel evidence that USP19 protects unstable PAH variants R241C and R243Q from rapid degradation.

## Results

### Generation and analysis of PAH protein mutants

**Figure 1:**
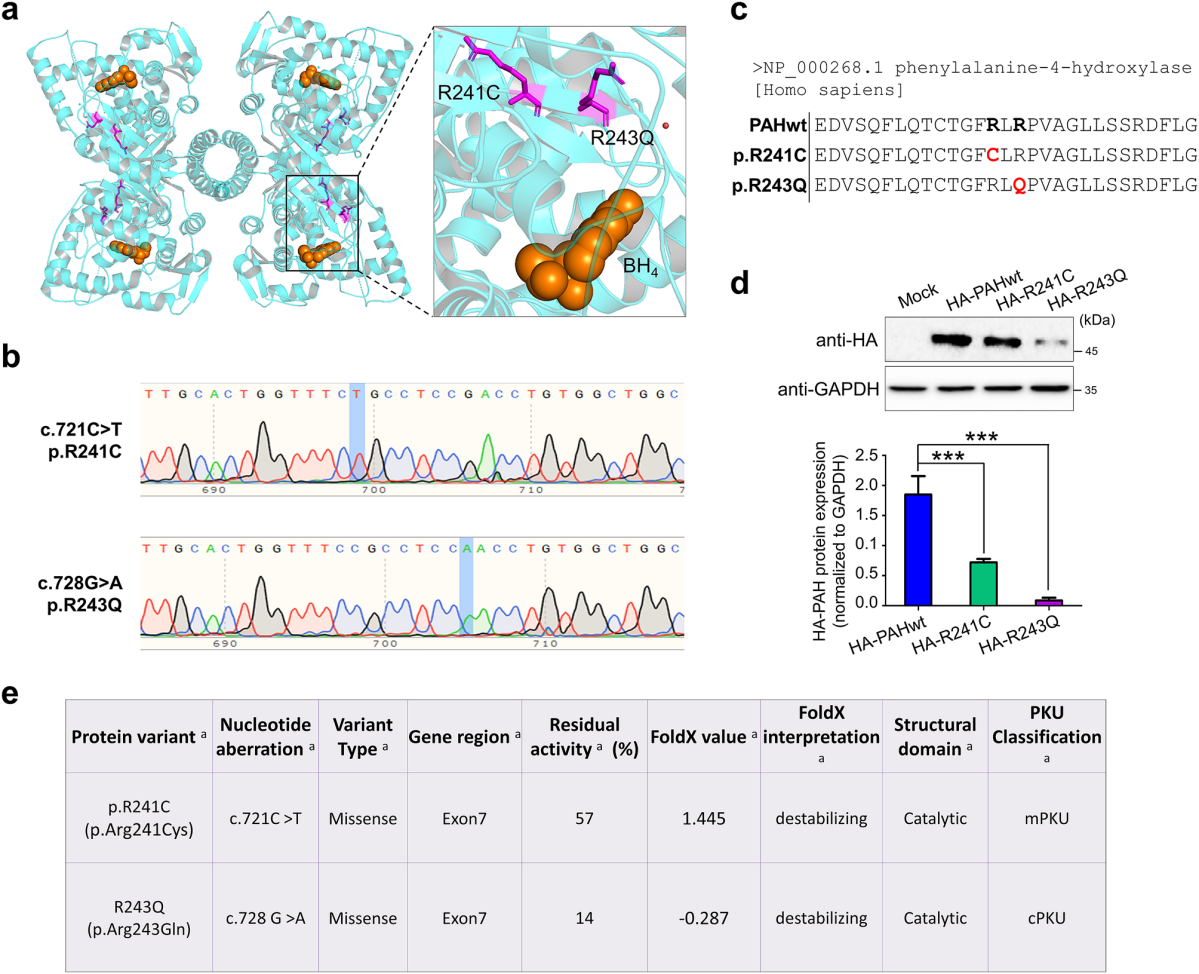
Generation of PAH variants by mutagenesis. (**a**) The crystal structure of the PAH tetramer in an auto-inhibited conformation (PDB code 6HYC) is presented as ribbon (cyan). The location of R241C and R243Q mutations in all four subunits is presented in magenta. BH_4_ cofactor is shown as orange spheres in all four subunits indicating the location of active site. An enlarged image of the mutation sites (R241C and R243Q) and cofactor BH_4_ in one of the subunits is shown on the right. The figure was generated using the PyMol program. (**b**) Sanger sequencing analysis of *PAH_*c.721C>T (R241C) and c.728 G>A (R243Q) showing point mutations on the *PAH* gene. (**c**) The amino acid substitutions for R241C and R243Q are represented. NCBI Reference Sequence: NP_000268.1. The red font denotes the amino acid change from arginine (R) to cysteine (C) in p.R241C and glutamic acid (Q) in p.R243Q variant. (**d**) Western blot analysis of PAHwt, R241C and R243Q variants. Band intensity was estimated using ImageJ software, normalized to GAPDH, and represented graphically. One-way ANOVA followed by Tukey’s post hoc test was used. Data are presented as the mean ± SD of three independent experiments (***P < 0.0005). (**e**) The PAH variants selected for this study. Data from ^a^PAHvdb (https://www.biopku.org) and the literature: ^b^^13^. Abbreviations: cPKU, classic phenylketonuria; mPKU, mild phenylketonuria.

Several mutations in the *PAH* gene have been studied, and most of them are missense mutations that are spread throughout the PAH protein. The R241C and R243Q mutations, which occur frequently in the Asian population, are considered in this study. The nucleotide aberrations c.721C>T and c.728 G>A in *PAH* exon 7 code for the p.Arg241Cys (p.R241C) and p.Arg243Gln (R243Q) mutations and are some of the most abundant mutations among PKU patients, with an average allele frequency of approximately 6% (9–14% in Mediterranean countries and the Middle East), with ~ 2% of patients homozygous for this mutation (up to 12% in Mediterranean countries and the Middle East)^9,21^.The PAH protein exists in two distinct conformations^22,23^, an auto-inhibited PAH illustrated in Fig. 1a and an activated PAH conformation (not shown). The auto-inhibited tetrameric structure of human PAH (hPAH) in complex with cofactor BH_4_ and the mutation sites (R241C and R243Q) located in the PAH catalytic domain are shown in Fig. 1a.

To generate the c.721C>T (R241C) and c.728 G>A (R243Q) mutations in PAHwt, we used site-directed mutagenesis. The oligonucleotides used to generate the PAH variants are described in Supplementary Table S1. The mutant clones were subjected to Sanger sequencing to confirm the point mutation. The sequencing results confirmed nucleotide aberrations at position 721, C>T, and position 728, G>A (Fig. 1b). The amino acid arginine was converted to cysteine at position 241 (R241C), and arginine was converted to glutamic acid at position 243 (R243Q) (Fig. 1c). We next performed an expression analysis of the two mutant proteins and the PAHwt protein. We transfected HEK293 cells with constant amounts of HA-tagged PAHwt, R241C, and R243Q and analyzed their expression levels by Western blotting. The expression of PAH was normalized to that of GAPDH. We found that both the R241C and R243Q variants were expressed at lower levels than PAHwt, particularly the R243Q variant, which showed a significant reduction compared with both R241C and PAHwt (Fig. 1d). These point variants have been reported to have residual enzymatic activity^24,25^ (https://www.biopku.org). Figure 1e represents the site of mutation in the *PAH* gene, residual in vitro enzyme activity, protein stability prediction (FoldX), and assignment to metabolic phenotypes of the *PAH* gene variations investigated in this study.

### PAH variants undergo proteasomal degradation

**Figure 2:**
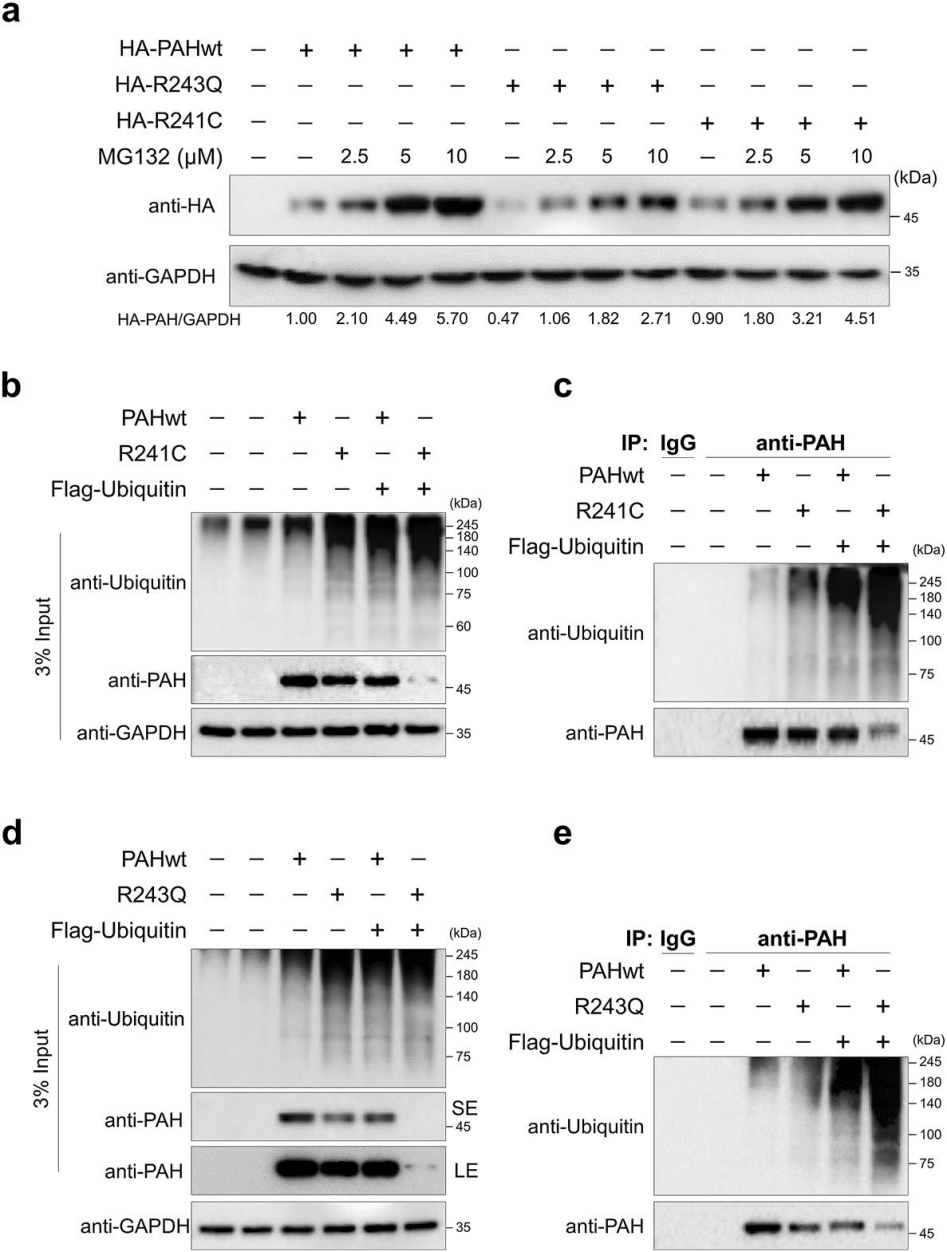
PAH variants are targets of proteasomal degradation. (**a**) The levels of R241C and R243Q proteins in HEK293 cells were determined by Western blotting after treatment with MG132 (2.5, 5, and 10 μM) for 12 h. Protein band intensities were estimated using the ImageJ software with reference to the GAPDH control. The band intensity for HA-PAH/GAPDH was represented below the blots. (**b-e**) HEK293 cells stably expressing PAHwt, R241C, or R243Q were transfected with Flag-ubiquitin to evaluate their ubiquitination status. Cells treated with IgG and cells transfected with empty vector were used as the negative controls. (**b**, **d**) represent the total cell extract used in the immunoprecipitation (input); (**c**, **e**) represent immunoprecipitation using anti-PAH antibody and immunoblotted with anti-ubiquitin and anti-PAH (SE, short exposure; LE, long exposure).

The proteasomal pathway is a major degradation pathway in cells that can degrade 80% of cellular proteins^26^. To confirm whether PAHwt, R241C, and R243Q proteins undergo protein degradation by the 26S proteasomal pathway, we transfected HEK293 cells with PAHwt, R241C, and R243Q variants and treated the cells with an increasing concentration of the MG132, a proteasomal inhibitor for 12 h. Our results indicate that the PAHwt and R241C variant proteins accumulated in a dose-dependent manner as the MG132 concentration increased (Fig. 2a). However, the R243Q PAH protein level was about twofold lower than the PAHwt accumulated at 10 μM concentration of MG132 treatment, suggesting that the R243Q PAH variant is highly susceptible to proteasomal degradation (Fig. 2a).

In addition to the proteasomal system, recent reports suggested that the PAH protein undergoes degradation through the autophagy pathway^10,17,18^. Thus, we sought to determine the effect of increasing the concentration of lysosomal inhibitor ammonium chloride (NH_4_Cl) on PAHwt, R241C and R243Q proteins. Autophagy inhibition was confirmed by accumulation of the autophagosome marker LC3-II, which is an overall indicator of autophagy impairment. NH_4_Cl treatment increased protein levels of the PAHwt, R241C, and R243Q variants compared to untreated cells (Supplementary Fig. S2), indicating that these variants might undergo degradation by an autophagy system. Since the main objective of this study was to analyze the deubiquitinating effect of USP19 on PAH variants R241C and R243Q, we expanded our research on UPS-mediated degradation of PAH variants.

Next, to investigate the ubiquitination of PAHwt and the mutant variants, HEK293 cells stably expressing PAHwt, R241C, and R243Q were transfected with Flag-tagged ubiquitin. Immunoprecipitation assays were then performed using PAH antibody, followed by Western blotting with specific endogenous antibodies against PAH and ubiquitin (Fig. 2b–e). Similarly, the ubiquitination of PAHwt and the mutant variants was cross-confirmed exogenously by co-transfecting HA-tagged PAHwt, R241C, R243Q, and Flag-ubiquitin. The cells were subjected to an immunoprecipitation assay using anti-HA antibody and immunoblotted with anti-Flag and anti-HA antibodies (Supplementary Fig. S3). Our data show that the R241C and R243Q variants had higher ubiquitination smears than PAHwt (Fig. 2b–e and Supplementary Fig. S3). Interestingly, the R243Q variant showed an increase in conjugated ubiquitin molecules compared with the R241C variant (Fig. 2c,e), suggesting that the misfolding-related instability of the R243Q variant resulted in increased ubiquitination and is susceptible to the proteasomal degradative system.

### USP19 interacts with PAH variants

**Figure 3:**
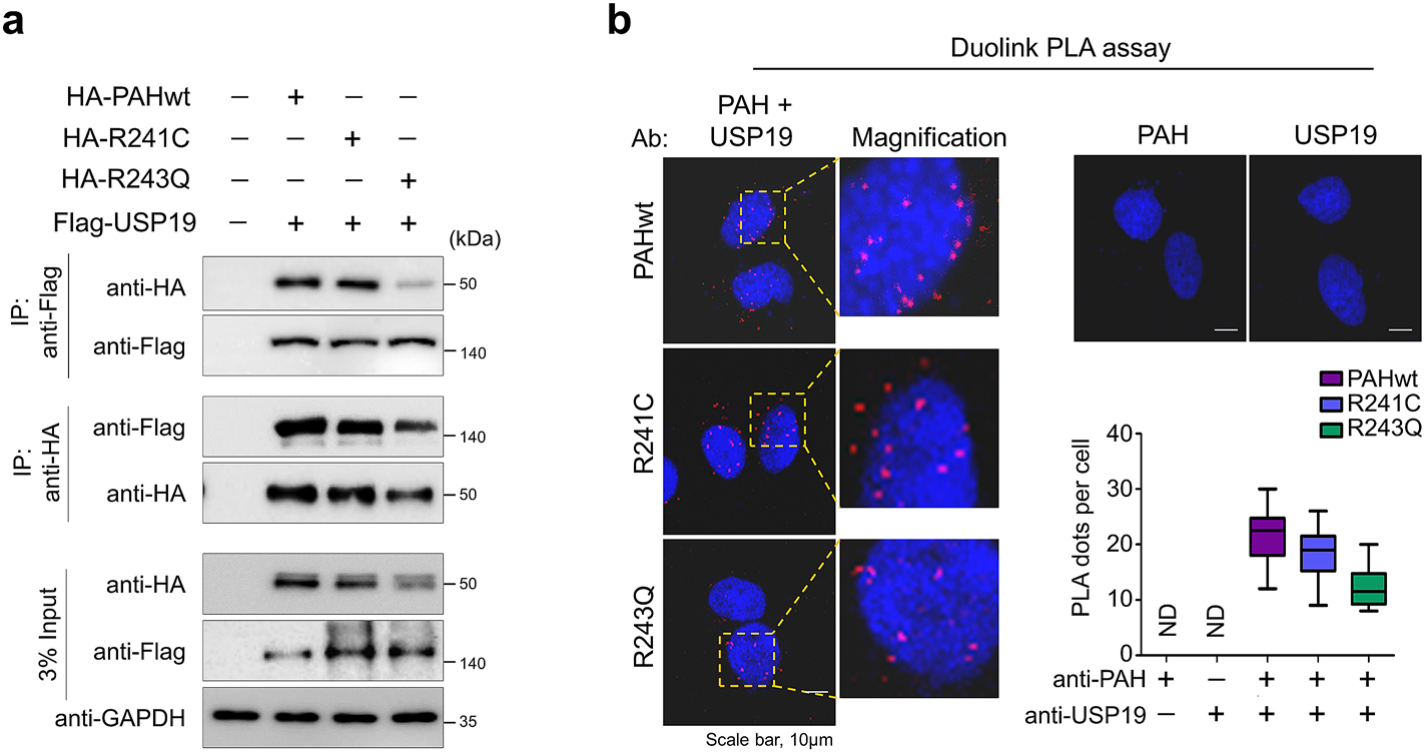
USP19 interacts with R241C and R243Q. (**a**) HEK293 cells were transfected with the indicated plasmids, followed by a co-immunoprecipitation assay to evaluate interactions between the PAH variants and USP19. Cells transfected with empty vector were used as the negative control. (**b**) HEK293 cells transfected with HA-tagged PAHwt, R241C, and R243Q were subjected to immunostaining using USP19 and PAH antibodies together (left panel). Cells stained with PAH or USP19 antibodies alone serve as negative control (right panel). DAPI was used to stain the cell nuclei, and the interaction was assessed using a Duolink PLA assay. Scale bar = 10 µm. Quantification of the PLA dots is shown as mean ± SEM.

To assess whether USP19 binds with the PAH variants, R241C and R243Q, we transfected HEK293 cells with PAHwt, R241C, and R243Q along with USP19, co-immunoprecipitated them using anti-Flag and HA antibodies, and conducted immunoblotting with reciprocal antibodies. The anti-Flag antibody immunoprecipitated USP19 along with PAHwt and the R241C and R243Q proteins. In a reciprocal immunoprecipitation, anti-HA antibody immunoprecipitated PAHwt and the PAH variants along with USP19, indicating that USP19 interacts with PAHwt and the R241C and R243Q proteins (Fig. 3a). Our results showed that the interaction between USP19 and R243Q was weaker than that between USP19 and the R241C variant (Fig. 3a). One reason could be the lower R243Q protein abundance for interaction with USP19 because of its rapid proteolysis by the proteasomal degradative system. Additionally, we performed a Duolink PLA assay to analyze the interaction between USP19 and PAHwt and the PAH variants. As shown in Fig. 3b, in situ USP19 and PAH interaction was observed in the form of PLA dots when USP19 and PAHwt or the PAH variants were immunostained together (Fig. 3b, left panel), but not when they were stained with either USP19 or PAH antibody alone (Fig. 3b, right panel). Thus our data suggest that USP19 interacts with PAHwt and the PAH variants.

### USP19 extends the half-life of PAH variant proteins through its deubiquitinating activity

**Figure 4:**
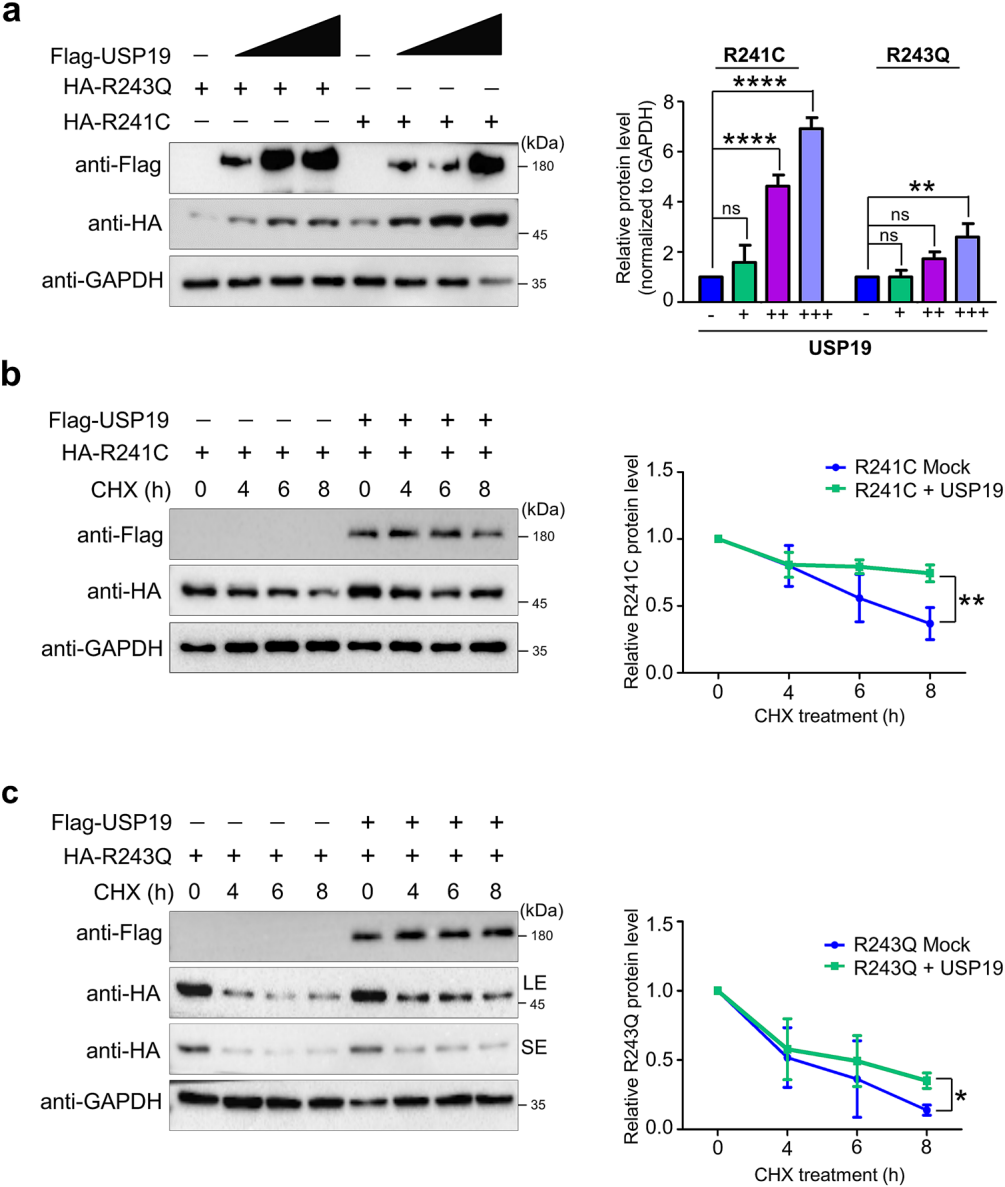
USP19 extends the half-lives of PAH variants. (**a**) HEK293 cells were transfected with PAHwt, R241C, or R243Q and an increasing concentration (0, 100, 250, 500 ng/mL) of USP19 and analyzed by Western blotting. (**b**) HEK293 cells were transfected with R241C and USP19, treated with CHX (150 μg/mL), harvested at different time points, and analyzed by Western blotting with the antibodies indicated. **c** HEK293 cells were transfected with R243Q and USP19, treated with CHX (150 μg/mL), harvested at different time points, and analyzed by Western blotting with the antibodies indicated (SE: short exposure; LE: Long exposure). The band intensities of R241C and R243Q from (**a**–**c**) were estimated using ImageJ software, normalized to GAPDH, and represented graphically. Data are presented as the mean and standard deviation of three independent experiments. Šídák's multiple comparisons test was used to evaluate the *P* value (*P < 0.05, **P < 0.005, ****P < 0.00005; ns denotes non-significant).

To examine the effect of USP19 on protein level of PAH variants, we transfected HEK293 cells with constant amounts of R241C and R243Q along with increasing concentrations of USP19 and analyzed by Western blotting. Our results demonstrate that USP19 offers dose-dependent increase in R241C and R243Q protein level (Fig. 4a). Next, we investigated the effect of USP19 on the protein turnover of the R241C and R243Q variants. To that end, we used the translation inhibitor CHX to examine the protein turnover from 0 to 8 h. We transfected HEK293 cells with R241C and R243Q proteins with and without USP19 and then treated the cells with CHX for 8 h. The half-lives of both R241C (Fig. 4b) and R243Q (Fig. 4c) were extended in the presence of USP19. Interestingly, the half-life of the highly unstable R243Q variant was significantly extended by USP19, suggesting that USP19 positively regulates the protein turnover of PAH variants.

**Figure 5:**
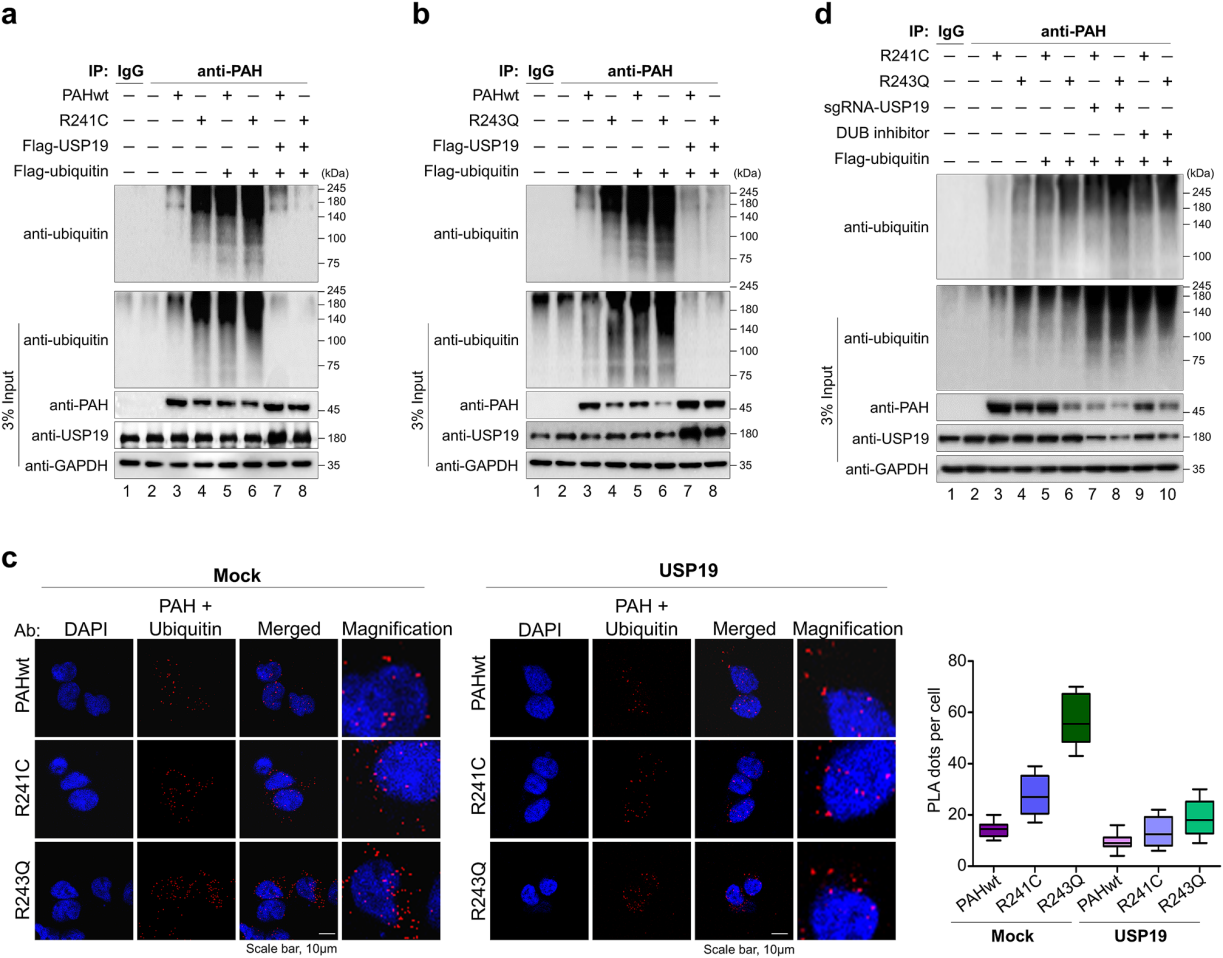
Deubiquitination of R241C and R243Q proteins by USP19. (**a**) The effects of USP19 overexpression on the ubiquitination and proteasomal degradation of PAHwt and R241C were determined. HEK293 cells stably expressing PAHwt and the R241C variant were transfected with Flag-ubiquitin and USP19 to analyze the ubiquitination status of PAHwt and R241C. Cells treated with IgG and cells transfected with empty vector were used as the negative controls. (**b**) The effects of USP19 overexpression on the ubiquitination and proteasomal degradation of PAHwt and R243Q were determined. HEK293 cells stably expressing PAHwt and the R243Q variant were transfected with Flag-ubiquitin and USP19 to analyze the ubiquitination status of PAHwt and R243Q. Cells treated with IgG and cells transfected with empty vector were used as the negative controls. (**c**) Duolink proximity ligation assay analysis of Ub-PAH expression was conducted in the presence and absence of USP19. DAPI was used to stain the nuclei. Scale bar = 10 µm. Quantification of the PLA dots is shown as the mean ± SEM. (**d**) The effects of sgRNA-mediated knockdown of *USP19* and the DUB inhibitor PR-619 on the ubiquitination of R241C and R243Q were analyzed after 48 h of treatment. Cells treated with IgG and cells transfected with empty vector were used as the negative controls.

Next, we examined the effect of USP19 on the ubiquitination level of PAHwt and its variants. HEK293 cells stably expressing PAHwt, R241C, and R243Q were transfected with Flag-ubiquitin and Flag-USP19. PAHwt, R241C, and R243Q were then immunoprecipitated using PAH antibody, followed by immunoblotting using ubiquitin- and USP19-specific antibodies. Our results show that both of the PAH variants displayed increased polyubiquitination smears compared with PAHwt, which was reduced by the deubiquitinating activity of USP19 (Fig. 5a,b, lane 5 and 6 vs. lane 7 and 8). To further validate those findings, we performed a Duolink PLA assay to determine the interaction between PAH and ubiquitin in the presence and absence of USP19. Our results demonstrate that USP19 overexpression reduced the interaction between ubiquitin and PAHwt and the PAH variants, compared with the mock control cells (Fig. 5c). Thus, USP19 has a key regulatory function on PAH variants by extending their half-lives, and preventing their degradation.

Next, we used the DUB inhibitor PR-619 and a previously validated sgRNA against *USP19* from our CRISPR-based DUB knockout library^27^ to transiently knock down *USP19* levels, and analyzed the ubiquitination status of PAH. The efficiency of sgRNA targeting *USP19* was analyzed on HEK293 cells stably expressing PAHwt by Western blot analysis. We observed that the protein levels of PAH were reduced when USP19 was depleted with sgRNA2 (Supplementary Fig. S4), hence we used sgRNA2 for further experiment. HEK293 cells stably expressing the R241C and R243Q variants were transfected with Flag-ubiquitin and the sgRNA targeting *USP19* or treated with the DUB inhibitor PR-619 (20 μM). The cells were then subjected to immunoprecipitation using PAH antibody and immunoblotted with ubiquitin antibody. We found that inhibiting USP19, either by sgRNA-mediated knockdown or by DUB inhibitor, prevented it from rescuing the ubiquitination of the two PAH variants (Fig. 5d, lane 7–10). Thus our results indicate that USP19 deubiquitinates PAHwt and PAH variants and rescues them from rapid proteasomal degradation.

### USP19 extends residual enzymatic activity of the PAH variants

**Figure 6:**
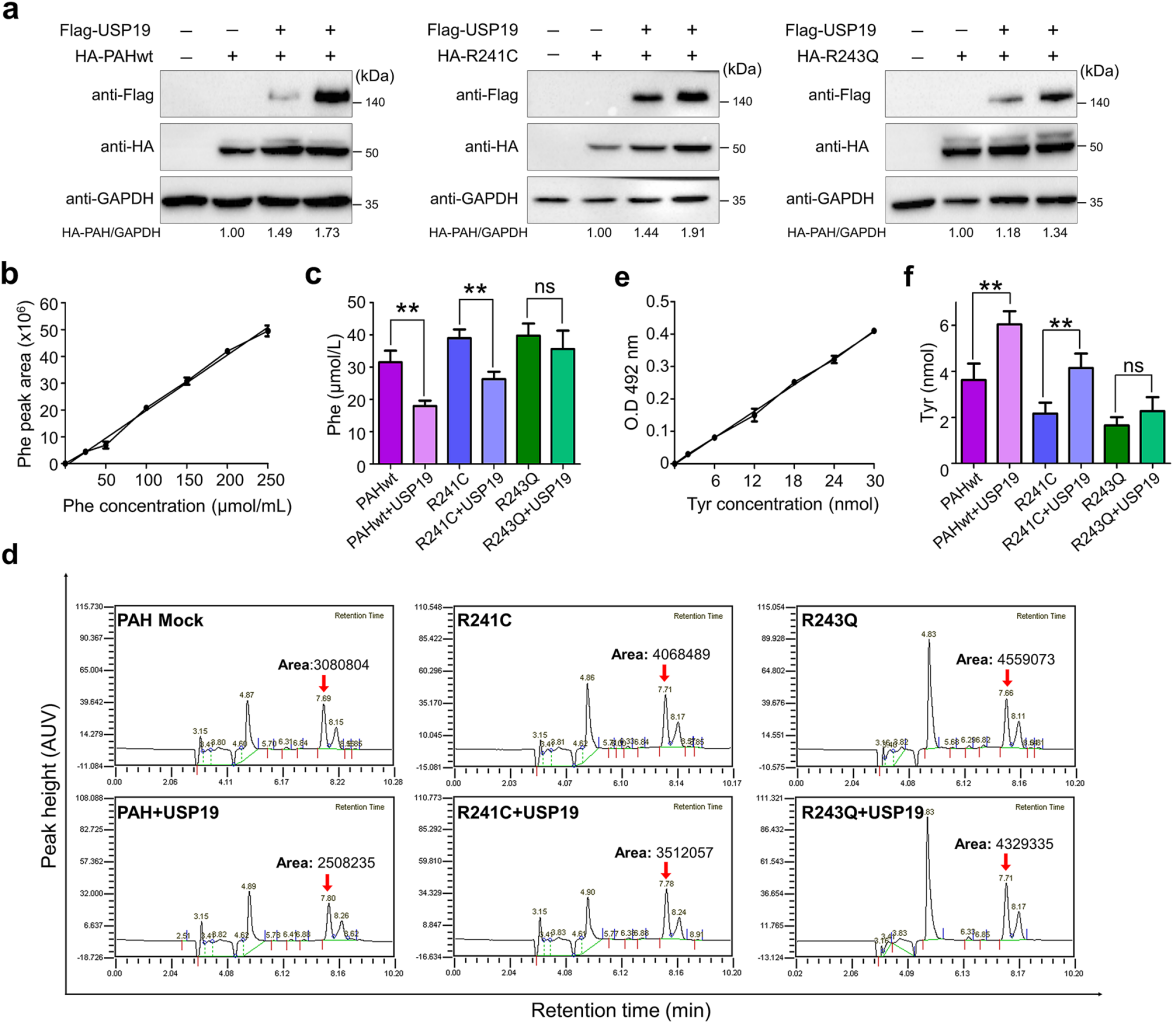
USP19 extends the residual enzymatic activities of R241C and R243Q. (**a**) Western blot analysis shows the transfection efficiency of the respective constructs used for further experiments. (**b**) A standard curve of Phe obtained from HPLC, representing an increasing concentration of Phe in µmol/mL, was plotted against the Phe peak area. (**c**) The residual activity was analyzed by HPLC by quantifying the amount of Phe metabolized by cells expressing R241C and R243Q in the presence of USP19. Data are presented as the mean ± SD of three independent experiments. One-way ANOVA followed by Tukey's multiple comparisons test was used (**P < 0.005; ns denotes non-significant). (**d**) The HPLC chromatogram of a PAH activity assay shows a Phe peak at a retention time of ~ 7.7 min. The Phe peak is indicated by a red arrow, and the measurement of the peak area is denoted at the top of the peak. (**e**) Standard curve of Tyr, representing an increasing concentration of Tyr in nmol, was plotted against the absorbance at OD 492 nm. (**f**) A tyrosine assay kit was used to analyze the residual activity of R241C and R243Q in the presence of USP19. Specific PAH activity is expressed as the amount of Tyr (nmol) produced by each sample. Data are presented as the mean ± SD of three independent experiments (**P < 0.005; ns, non-significant).

Because some PAH variants exhibit a certain amount of residual activity, we investigated whether USP19 overexpression could extend the residual enzyme function of the R241C and R243Q PAH variants. We co-transfected HEK293 cells with PAHwt, R241C, or R243Q and increasing concentrations of USP19 and analyzed the results by Western blotting. In the presence of USP19, the protein levels of R241C and R243Q variant were increased up to 1.9-fold and 1.3-fold, respectively (Fig. 6a). The functionality of PAHwt, R241C, and R243Q was tested by quantifying the Phe levels using HPLC in the presence of USP19. A standard curve was plotted using a known amount of Phe in triplicate and used to determine the Phe concentration in the samples (Fig. 6b). In the presence of USP19, the percentage of Phe in cells expressing R241C and R243Q was reduced by about 1.7-fold and 1.1-fold respectively (Fig. 6c,d). In a reciprocal manner, we confirmed the amount of Tyr produced by R241C and R243Q using a tyrosine kit. A standard curve was plotted using a known amount of Tyr in triplicate and used to determine the Tyr concentration in the samples (Fig. 6e). Consistent with our HPLC results, the metabolism of Phe in cells expressing R241C and R243Q was increased by USP19. In the presence of USP19, the total amount of Tyr product in cells expressing R241C and R243Q was increased by about 1.6-fold and 1.3-fold, respectively (Fig. 6f). Overall, our results suggest that USP19 increases the cellular protein level of PAH variants by extending their half-lives, which results in increased Phe metabolism.

## Discussion

PKU is a complex metabolic disorder caused by deficiency of PAH activity. Our understanding of PAH structure was recently advanced by the identification of the crystal structure for the full-length hPAH protein^22,23^. The multidomain PAH protein exists in two distinct conformations, an auto-inhibited conformation and a distinct activated conformation. The transition from auto-inhibited structure to activated PAH conformation is dependent on Phe binding to an allosteric site of the N-terminal regulatory domain, causing large movements and dimerization of the regulatory domain exposing active sites^28^. With the rise in Phe level, equilibrium shifts from auto-inhibited to activated PAH conformation, maintaining Phe below a neurotoxic level^23,29^. More than 1,500 mutations have been identified in the *PAH* gene according to the locus-specific database, PAHvdb (http://www.biopku.org/pah/), among which over 500 mutations are associated with PKU^30–32^. Certain PKU-associated mutants have been demonstrated to result in protein-folding defects (e.g. R261Q, p. L249P)^18,33^, impairment of PAH catalytic activity (e.g. H170Q, P275L, A300S)^34^ or to affect conformational stability (e.g. G46S, R408W, I65S)^34–36^. In addition, PKU-associated mutations might cause inability in the transition from auto-inhibited PAH to the activated conformation^29^. Therefore, diverse therapeutic approaches have to be investigated to address the breadth of PKU disease-causing mutants.

In this study, we considered PAH variants R241C and R243Q, which are prevalent in the Asian population and are associated with both mild and severe PKU, with residual activity of 25% and 12.8%, respectively^11^. A report suggested that high correlation exists between the structural destabilization of PAH variants and PKU disease severity^14^. Moreover, the misfolding-associated mutation in *PAH* acts as a trigger for rapid protein degradation, leading to insufficient availability of cellular PAH^10,18,37,38^. Likewise, we observed that the expression of both R241C and R243Q PAH variants was lower than that of the PAHwt protein. The R243Q variant in particular displayed about sixfold lower protein level than PAHwt (Fig. 1d), which was in line with previous reports^14,25^. Interestingly, we found that reduced levels of PAH variants were caused by ubiquitin-mediated proteasomal degradation, while treatment with proteasomal inhibitor was able to rescue PAH protein from degradation (Fig. 2).

The autophagy-lysosomal pathway is another major pathway that mediates the degradation of misfolded PAH variants. Cross-talk between UPS and autophagy pathways is critical for maintaining cellular proteostasis^18,39,40^. The PAH amyloid-like aggregates in PAH-R261Q mouse liver co-localize with autophagy markers such as P-p62 and LC3, which is a strong evidence of selective autophagy degradation of PAH-R261Q^18^. In addition to UPS-mediated degradation of R241C and R243Q variants (Fig. 2a), we observed that dose-dependent treatment of lysosomal inhibitor gradually increased protein levels of R241C and R243Q variants (Supplementary Fig. S2), suggesting that degradation of these variants is also associated with an autophagy pathway.

Chaperones and ubiquitin are important nodes mediating crosstalk between the proteasomal and autophagic degradation pathways. DNAJC12, a co-chaperone of HSP40, interacts with ubiquitinated PAH and might play an important role in HSP70-mediated folding or degradation of its client protein. Patients with biallelic mutations in the *DNAJC12* gene were reported to exhibit HPA disorders with reduced PAH levels and activity^41^. The function of DNAJC12 was mutation-specific, where it increased the protein levels and enzymatic activity of a few mutant PAH variants such as p.L48S, p.I65T and p.R261Q, while reducing the protein levels and enzymatic activity of other PAH variants such as p.E280K, p.L348V, and p.V388M^41^. Moreover, increased cellular levels of PAH variants reduced disease severity as observed in the case of the F39L variant^42^. Therefore, we initiated this study to investigate whether USP19, a previously identified DUB for PAHwt^20^, can increase cellular levels of PAH variants through deubiquitinating activity.

Notably, USP19 is an endoplasmic reticulum (ER)-specific DUB and participates in the export of misfolding-associated protein, which are associated with neurodegenerative diseases^43^. USP19 is frequently increased during the unfolded protein response and protects ER-associated degradation of substrates such as CFTR ΔF508 from proteasomal degradation through its deubiquitinating activity^44^. Interestingly, USP19 is the first DUB found to have activity that is promoted by HSP90, a chaperone protein. The N-terminal region of USP19 contains two CS/P23 domains and interacts with HSP90 and enhances substrate recognition^45,46^. The USP19-HSP90 machinery has been proposed to be involved in regulating the stability of huntingtin and ataxin-3 proteins, playing a crucial role in the protein triage decision associated with the aggregation and protein degradation^46^. In contrast, it is possible that DUBs enhance the refolding efficacy of chaperone and thus cooperate with chaperones and promote stabilization of their target substrate. Previously, we demonstrated that USP19 extends the half-life of PAHwt protein by preventing its proteasomal degradation^20^. In the current study, we investigated the role of USP19 on variant PAH protein turnover and its metabolic function.

We observed that USP19 positively regulates and binds with the R241C and R243Q PAH variants similar to the PAHwt protein (Fig. 3a). Additionally, an in situ Duolink PLA assay also showed interaction between USP19 and R241C (Fig. 3). However, the R243Q variant, which is associated with severe PKU, showed relatively less interaction with USP19 (Fig. 3a,b), possibly due to the lower abundance of R243Q protein levels in the cell. Next, we showed that the R243Q variant undergoes accelerated degradation, with a shorter half-life than the R241C variant (Fig. 4b,c). To support that finding, we demonstrated that R243Q was highly ubiquitinated compared with both R241C and PAHwt (Figs. 2, 5) and hence displayed lower protein levels. Deubiquitinating enzymes can specifically counteract the ubiquitination of their substrate and have therapeutic value by regulating protein stability, aggregation, and degradation in diseases such as cystic fibrosis, Huntington's disease, and Parkinson’s disease^47–51^. Here, we demonstrated that USP19 deubiquitinated and restored sufficient amounts of PAH variant enzymes in cells by extending the half-lives of both R241C and R243Q (Figs. 4, 5). In contrast, on transient knockdown of *USP19*, both PAH variants were rapidly degraded, demonstrating the specificity of USP19’s deubiquitinating activity on PAH variants (Fig. 5d). Because these disease-linked PAH variants have shorter half-lives than PAHwt, an insufficient amount of cellular PAH protein is available, increasing disease severity^14^. Therefore, we investigated the functional consequence of USP19-mediated increase in the protein levels of the PAH variants on their metabolic function. Our results indicate that USP19 promotes Phe metabolism, which was quantified by findings of reduced Phe and increased product Tyr in HPLC and colorimetric assays (Fig. 6). The Phe metabolism exhibited by R241C was improved by USP19, but USP19 had only a subtle effect on protein level and Phe metabolism of the R243Q variant (Fig. 6), suggesting that the function of USP19 is dependent on the variant. Altogether, to develop better therapies for PKU-disease, it is necessary to further investigate the regulatory mechanisms of USP19 alone or in combination with chaperones to elevate cellular protein levels in PAH variants such as F39l, P122Q, F161S, and I65T, which are all susceptible to rapid proteasomal degradation^14^.

## Materials and methods

### Cell culture and treatments

Human embryonic kidney (HEK293) cells (Korean Cell Bank, Seoul, Korea) were cultured at 37 °C in a humidified atmosphere with 5% CO_2_ in Dulbecco’s modified Eagle’s medium (GIBCO BRL, Rockville, MD, USA) supplemented with 10% fetal bovine serum (GIBCO BRL) and 1% penicillin and streptomycin (GIBCO BRL). One day before transfection, the cells were seeded in a 6-well culture dish, and then about 1 μg of HA-PAHwt, HA-R241C, and HA-R243Q plasmids were added to each well for transfection. The concentration ranging from 100 to 500 ng of Flag-USP19 was transfected, depending on the experiment. Untransfected HEK293 cells were used as the control. Polyethyleneimine (Polysciences, Warrington, PA, USA) was used according to the manufacturer’s protocol to transfect the HEK293 cells. After 48 h, the cells were harvested. For the CHX assay, cells were transfected with the indicated constructs and incubated for two days. After 48 h of transfection, the cells were treated with CHX (150 μg/mL) and harvested at various time points. For the proteasomal or lysosomal inhibition, cells were transfected with indicated plasmid. After 48 h of transfection the cells were treated with MG132 for 12 h or ammonium chloride (NH_4_Cl) for 24 h respectively.

### Plasmids, antibodies, and reagents

HA-tagged PAH and the pLVX-IRES-ZsGreen1-PAHwt construct were kindly provided by Prof. Shen Nan (University Children’s Hospital, Heidelberg, Germany)^33,52^. PAH R241C and R243Q were generated in HA-tagged PAH and pLVX-IRES-ZsGreen1-PAHwt constructs by site-directed mutagenesis using the primers indicated in Supplementary Table S1. Flag-tagged USP19 and Flag-tagged ubiquitin were purchased from Addgene (Watertown, MA, USA). The following primary antibodies were used in our study: mouse monoclonal antibody against the HA tag (sc-7392, 1:1000), GAPDH (sc-32233, 1:1000), ubiquitin (SC-8017, 1:1000) and rabbit polyclonal antibody against ubiquitin (sc-9133, 1:500). Protein A/G Plus Agarose beads (sc-2003) were purchased from Santa Cruz Biotech (Dallas, TX, USA). Mouse anti-PH8 antibody (MAB5278, 1:1000, Merck Millipore, Kenilworth, NJ, USA), rabbit polyclonal antibodies against USP19 (Cat no. #25,768–1-AP, 1:1000, Proteintech, Rosemont, IL, USA), rabbit polyclonal antibody against LC3B (Cat No. #2775S, Cell signaling technology, Denvers, MA, USA) and mouse monoclonal antibody against the Flag tag (Anti-DDDDK-tag, M185-3L, 1:1000, MBL Life Science, Woburn, MA, USA) were used. In addition, we used IP lysis buffer (Cat. no. #87787; Thermo Fisher), cell lysis buffer (Cat. no. #R2002, Biosesang), and a protease inhibitor cocktail (Cat. no. #11836153001, Roche, South San Francisco, CA, USA), the proteasomal inhibitor MG132 (Cat. no. #S2619, Selleckchem, Houston, TX, USA), the protein translation inhibitor CHX (Cat no. #239765, Merck, Kenilworth, NJ, USA), the DUB inhibitor PR-619 (Cat. no. #S7130, Selleckchem, Houston, TX, USA) and Lysosomal inhibitor NH_4_Cl (Sigma-Aldrich, St. Louis, MO, USA).

### Stable expression of PAH and variants in HEK293 cells

To generate HEK293 cells that stably expressing PAHwt, R241C, and R243Q, we used the pLVX-IRES-ZsGreen1 plasmid encoding PAHwt, R241C and R243Q for lentiviral production. One day prior to transfection, 1 × 10^6^ HEK293 cells were seeded and cultured in a 100 mm culture dish. The HEK293 cells were then co-transfected with pLVX-IRES-ZsGreen1 plasmids encoding PAHwt, R241C, or R243Q and packaging vectors (pLP1, pLP2 and pLP-VSVG) in a 4:1:1:1 ratio. Cell supernatants were harvested 48 h after transfection and either used to infect cells or stored at − 80 °C. Cells were infected for 6 h with the lentiviral supernatants diluted 1:1 with normal culture medium in the presence of 10 μg/mL of polybrene (Sigma-Aldrich) to obtain stable HEK293 cell lines expressing PAHwt or a PAH variant. The transduction efficiency of pLVX-ZsGreen1 plasmid encoding PAHwt, R241C and R243Q was checked after 72 h (Supplementary Fig. S1a and S1b). The cells transduced with empty vector were used as control.

### Immunoprecipitation

HEK293 cells were lysed with IP lysis buffer containing 150 mM sodium chloride, 1 mM EDTA 25 mM, Tris–HCl (pH 7.4), 1% NP-40, 5% glycerol, 1 mM PMSF, and protease inhibitor cocktail for 20 min. The cell lysates were incubated with the indicated antibodies at 4 °C overnight and immunoprecipitated for 2–4 h with 20 μL of Protein A/G Plus Agarose beads (Santa Cruz Biotechnology) with constant agitation at 4 °C. The beads were washed thrice with lysis buffer and re-suspended in 2X denaturing SDS sample buffer (Cat. no. S3401, Sigma-Aldrich)): 5X SDS sample loading buffer containing 4% SDS, 10% 2-mercaptoethanol, 20% glycerol, 0.004% bromophenol blue, and 0.125 M Tris–HCl (pH 6.8). The samples were boiled at 95–100 °C for 5 min, followed by immunoblotting. For the binding assay, mouse IgG (Cat. No. #18-8817-33, Rockland, Philadelphia, Pennsylvania, USA), a light chain–specific secondary antibody, was used to prevent interference from heavy immunoglobulin chains.

### Duolink proximity ligation assay

A Duolink in situ proximity ligation assay (PLA) kit (DUO92101, Sigma-Aldrich) was used to detect the interaction between PAH and USP19 and to evaluate the ubiquitination status of PAH. HEK293 cells were fixed at room temperature in 4% paraformaldehyde for 10 min and blocked using 1X blocking solution. The cells were then incubated at 4 °C overnight with primary antibodies targeting PAH, USP19, or ubiquitin, followed by incubation with PLA probes at 37 °C for 1 h. After washing them three times, we added ligation-ligase solution and incubated them at 37 °C for 30 min. Next, the slides were incubated with amplification-polymerase solution at 37 °C in the dark for 100 min. Finally, the cells were stained with mounting medium containing DAPI. A Leica fluorescence microscope (Leica, DM 5000B; Leica CTR 5000; Wetzlar, Germany) was used to capture the fluorescence images.

### PAH activity assay

To measure PAH enzyme activity, transfected cells were harvested and lysed by three freeze–thaw cycles in lysis buffer (0.25 mol/L sucrose, 1X PBS, pH 7.2) containing a protease inhibitor. The samples were centrifuged at 15,800 g for 20 min at 4 °C to obtain clear lysates and determine PAH activity, as previously described^53^. Briefly, 120 μg of total protein was pre-incubated with 1 mmol/L of l-Phe (P17008, Sigma, St. Louis, MO, USA) and 2 µg of catalase (C1345, Sigma) in 0.1 mol/L Na-HEPES buffer (pH 7.0, T&I, BHE-9000, Gangwon, Korea) for 5 min followed by a 1 min incubation with 10 mM ferrous ammonium sulfate. The reaction was initiated by adding 200 µmol/L of BH_4_ (Cat no #T4425, Sigma) in 5 mM DTT (Cat no. #10197777001, Sigma) and allowed to proceed for 30 min at 25 °C. The reaction was stopped by adding 50 µL of 2% (*w/v*) acetic acid in ethanol. All concentrations mentioned refer to the final concentration in a 100 µL reaction mixture. The amounts of Tyr were determined using a tyrosine assay kit (Cat no. #ab185435) according to the manufacturer’s protocol. A standard curve for Tyr was used to determine the Tyr concentration in the samples (Fig. 6e).

For the HPLC–UV analysis, a Phenomenex EZ:Faast™ kit was used to prepare the samples. The amount of Phe was determined using an HPLC system with a UV detector at a wavelength of 210 nm. Acetonitrile (94:6 v/v), 20 mmol/L sodium acetate buffer (pH 6.5), and a Shiseido Capcell Pak MF C_8_ analytical column (4.6 mm × 150 mm) were used for the chromatography. The flow rate was 1 mL/min, and the sample injection volume was 20 µL. A standard curve for Phe was used to determine the Phe concentration in the samples (Fig. 6b). The chromatograms represent the peak height (in arbitrary units, AUV) against the retention time (Fig. 6d).

### Statistics

The statistical analysis was conducted using GraphPad Prism 9 (GraphPad Software, Inc. San Diego, CA, USA), and the results are presented as the mean ± standard deviation of three independent experiments. One-way ANOVA and paired t testing were used to analyze the data, and multiple comparisons among groups were performed with Tukey’s post hoc test or Šídák's multiple comparisons test. For comparisons between two groups, two-way ANOVA was used to analyze the data (*P < 0.05, **P < 0.005, ***P < 0.0005, ****P < 0.00005, and ns denotes non-significant).

### Consent to participate

All individual authors included in the study provide consent for publication. The authors are responsible for the correctness of the statements provided in the manuscript.

## Supplementary Information

Supplementary Information.

## Data Availability

All data generated or analyzed during this study are included in this published article (and its Supplementary Information files).

## Abbreviations

PAH: Phenylalanine hydroxylase
PKU: Phenylketonuria
PTM: Post translational modification
UPS: Ubiquitin–proteasome system
DUB: Deubiquitinating enzyme
APC/C: Anaphase-promoting complex/cyclosome
Cdh1: Cell division cycle 20 related protein 1
HEK293: Human embryonic kidney 293
NH_4_Cl: Ammonium chloride
CHX: Cycloheximide
HPLC–UV: High performance liquid chromatography–ultraviolet
