# Supplementary material for: Deubiquitinase USP19 extends the residual enzymatic activity of phenylalanine hydroxylase variants
Source: Sci Rep. 2022 Aug 20;12:14243. doi: 10.1038/s41598-022-18656-0 (PMC9392723; doi:10.1038/s41598-022-18656-0)
Supplement: Supplementary file 1 — Supplementary Information. [file 41598_2022_18656_MOESM1_ESM.docx]

***Supplementary File***

**Deubiquitinase USP19 extends the residual enzymatic activity of phenylalanine hydroxylase variants**

Neha Sarodaya^1†^, Apoorvi Tyagi^1†^, Hyun-Jin Kim^2^, Ju-Seop Kang^2^, Vijai Singh^3^, Seok-Ho Hong^4^, Woo Jin Kim^5^, Kye-Seong Kim^1,6*^, and Suresh Ramakrishna^1,6*^

^1^ Graduate School of Biomedical Science and Engineering, Hanyang University, Seoul, South Korea

^2^ Department of Pharmacology, College of Medicine, Hanyang University, Seoul, South Korea

^3^ Department of Biosciences, School of Science, Indrashil University, Rajpur, Mehsana, Gujarat, India

^4^ Department of Internal Medicine, School of Medicine, Kangwon National University, Chuncheon, South Korea

^5^ Department of Internal Medicine and Environmental Health Center, Kangwon National University Hospital, Kangwon National University School of Medicine, Chuncheon, South Korea

^6^ College of Medicine, Hanyang University, Seoul, South Korea

^†^ These authors have contributed equally: Neha Sarodaya and Apoorvi Tyagi

***Corresponding authors**

SR (E-mail: [suri28@hanyang.ac.kr](mailto:suri28@hanyang.ac.kr), [suresh.ramakris@gmail.com](mailto:suresh.ramakris@gmail.com));

KSK (E-mail: ks66kim@hanyang.ac.kr)

**Table of Contents**

**Supplementary Figure S1:** Transduction efficiency of pLVX-ZsGreen1 plasmid coding PAHwt, R241C and R243Q in HEK293 cells.

**Supplementary Figure S2:** The effect of lysosomal inhibitor on PAHwt and PAH variants R241C and R243Q.

**Supplementary Figure S3:** PAH variants are targets of proteasomal degradation.

**Supplementary Figure S4:** The effect of sgRNA targeting *USP19* on PAHwt protein.

**Supplementary Figure S5:** Original uncropped Western blot images with ladder/marker

**Supplementary Table S1:** Oligonucleotides used for generating PAH variant by site directed mutagenesis.

**
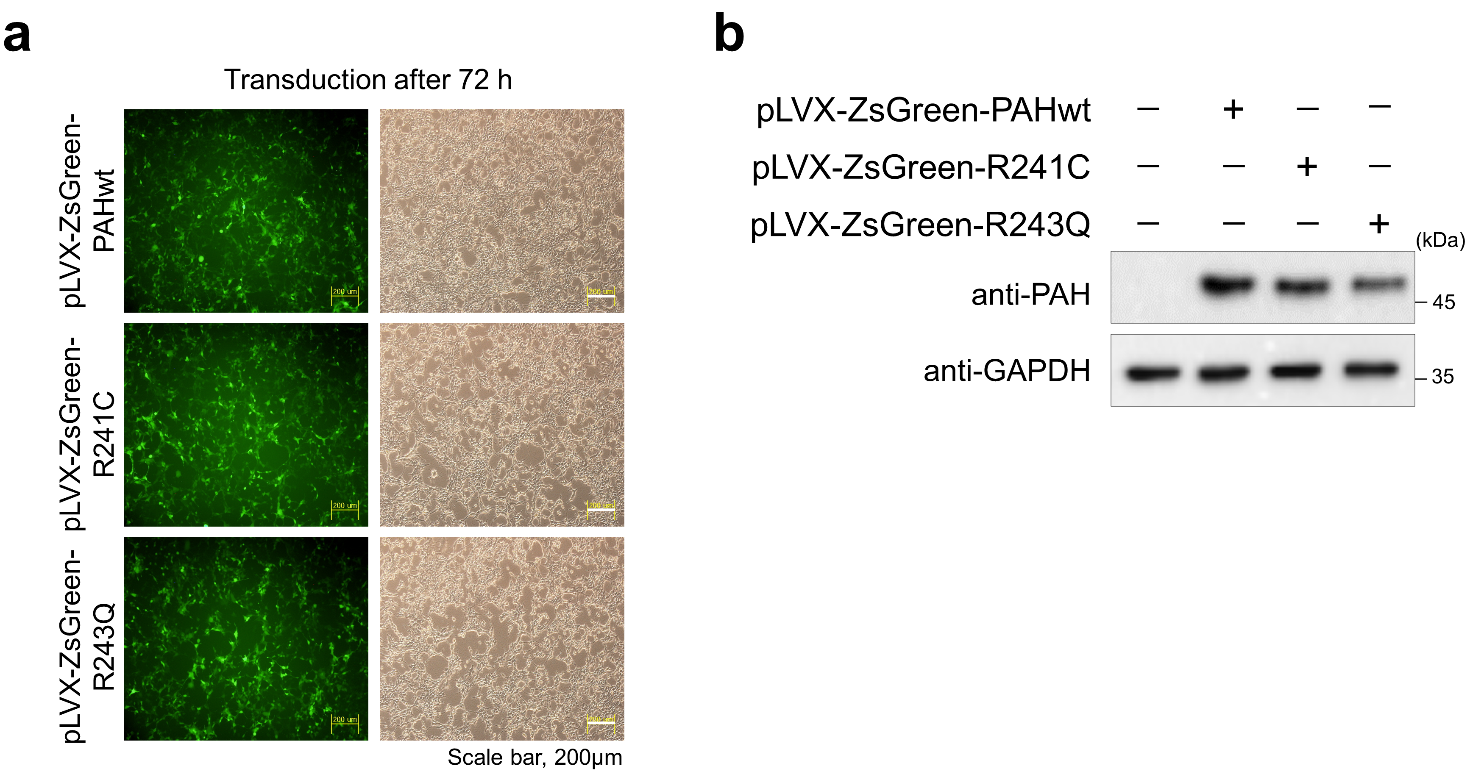
**

**Supplementary Figure S1:** Transduction efficiency of pLVX-ZsGreen1 plasmid coding PAHwt, R241C and R243Q in HEK293 cells. **a** Transduction efficiency of pLVX-ZsGreen1 plasmid coding PAHwt, R241C and R243Q in HEK293 cells after 72h (Scale bar, 200µm). **b** HEK293 cells stably expressing PAHwt, R241C and R243Q was validated by Western blot analysis.


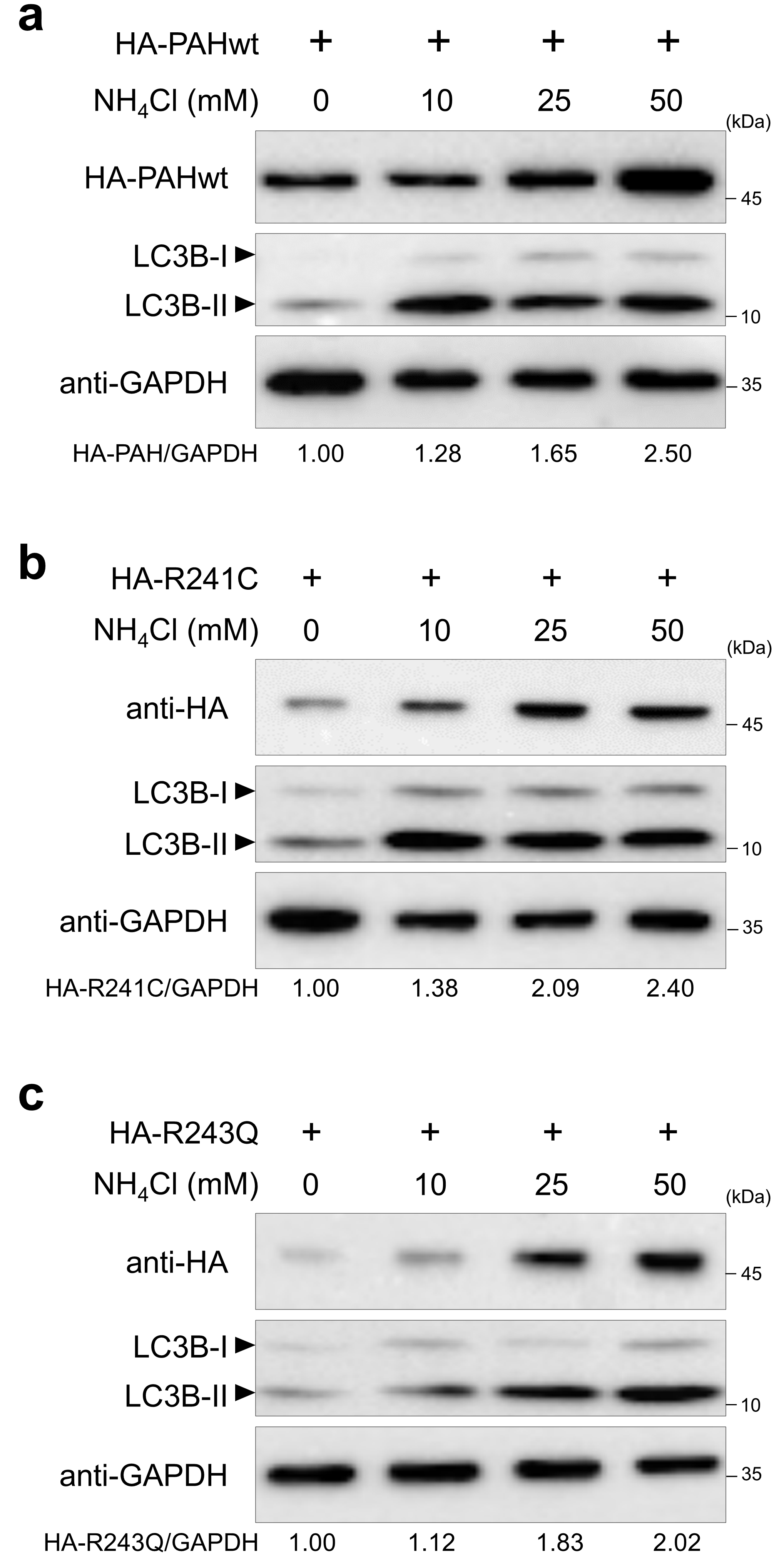


**Supplementary Figure S2:** The effect of lysosomal inhibitor on PAHwt and PAH variants R241C, R243Q. **a-c** HEK293 cells transfected with PAHwt, R241C or R243Q were treated with the indicated concentration of ammonium chloride (NH_4_Cl) for 24 h and analyzed by Western Blotting.


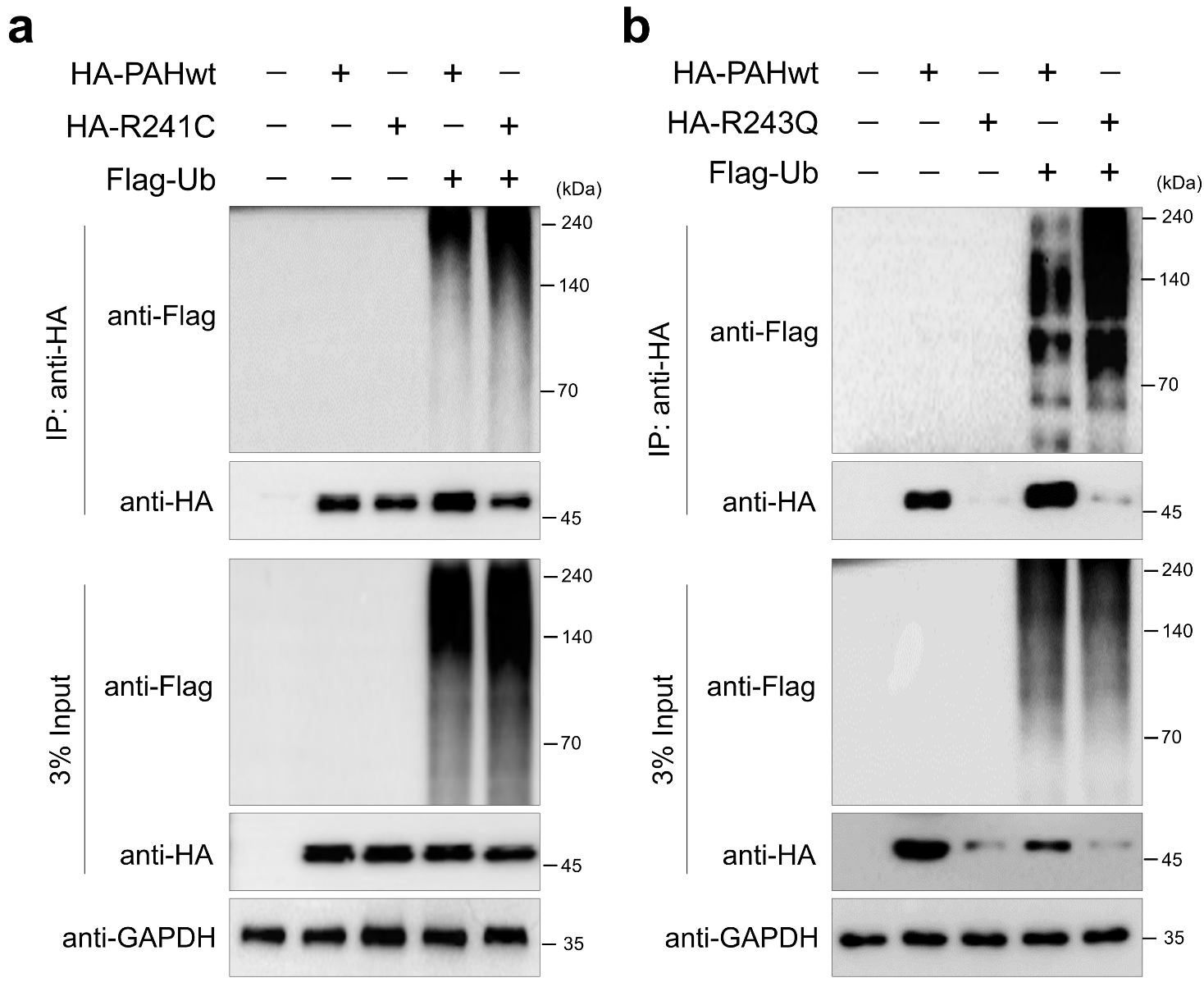


**Supplementary Figure S3:** PAH variants are targets of proteasomal degradation. **a** HEK293 cells were transfected with HA-tagged PAHwt, R241C and Flag-ubiquitin to evaluate the ubiquitination status of PAH variants. **b** HEK293 cells were transfected with HA-tagged PAHwt, R243Q and Flag-ubiquitin to evaluate the ubiquitination status of PAH variants.

**
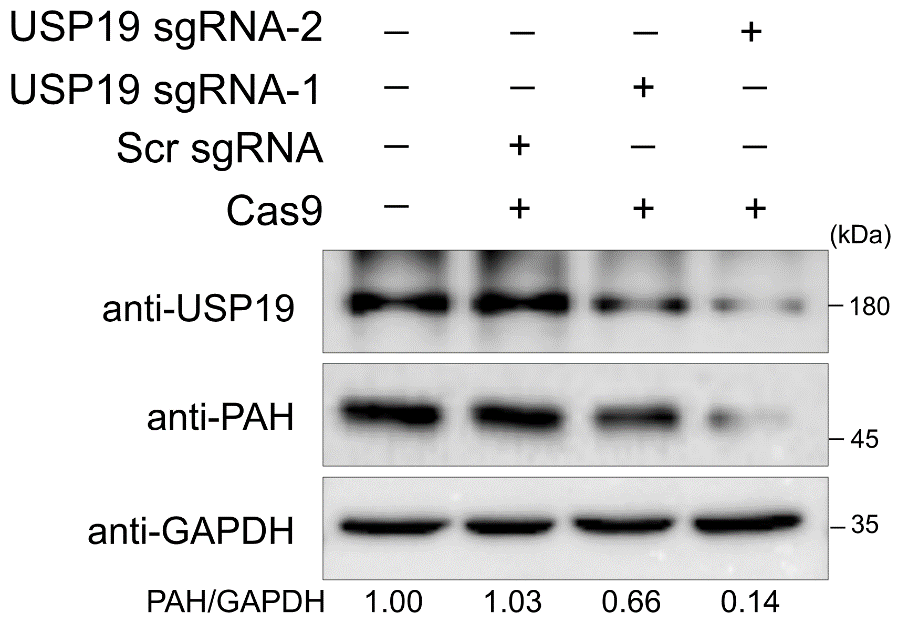
**

**Supplementary Figure S4:** The effect of sgRNA targeting *USP19* on PAHwt protein. The efficiency of sgRNA targeting *USP19* was analyzed on HEK293 cells stably expressing PAHwt by Western blot analysis.

**Supplementary Figure S5:** Original uncropped Western blot images with Ladder/ marker

**
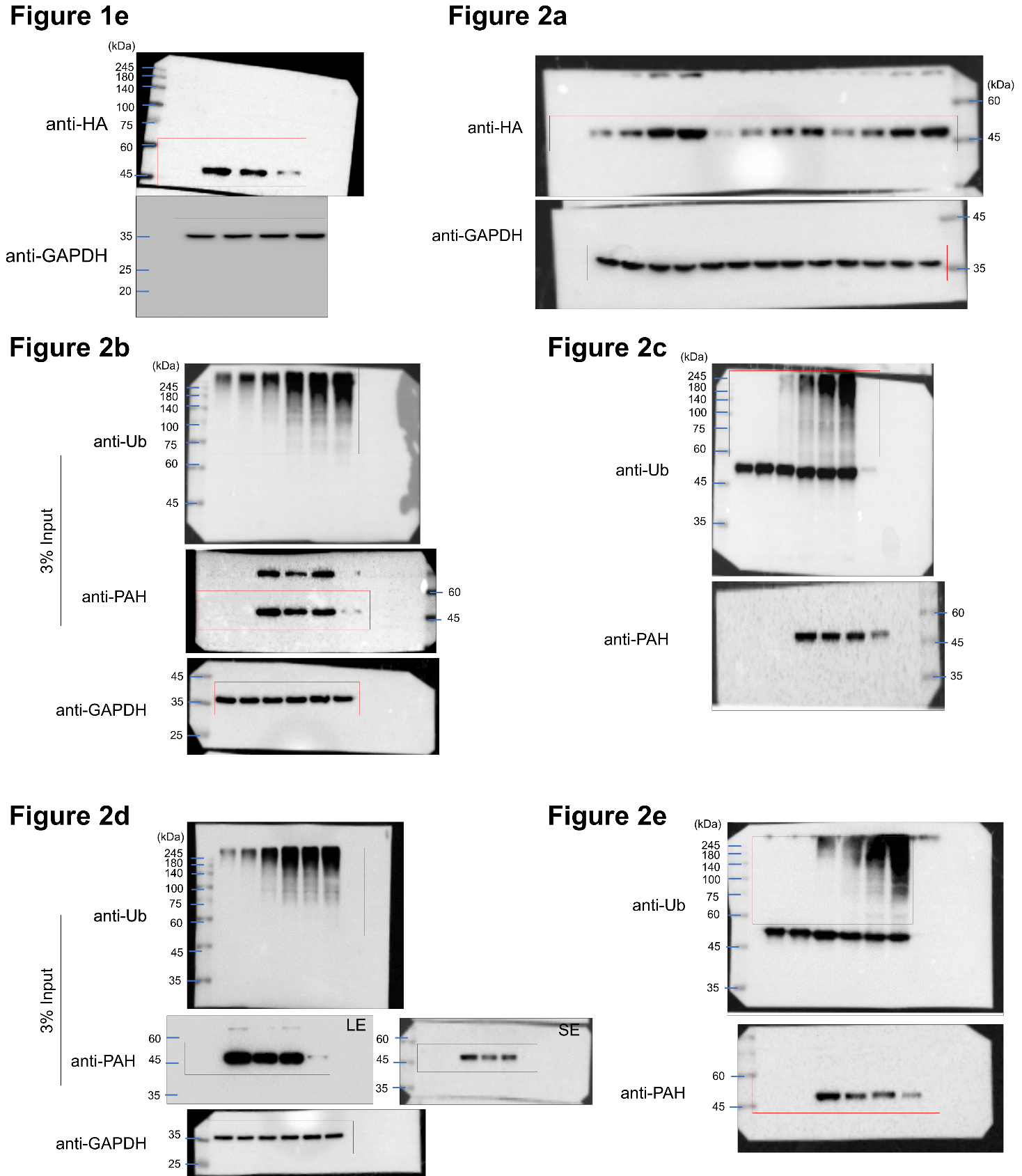
**

**
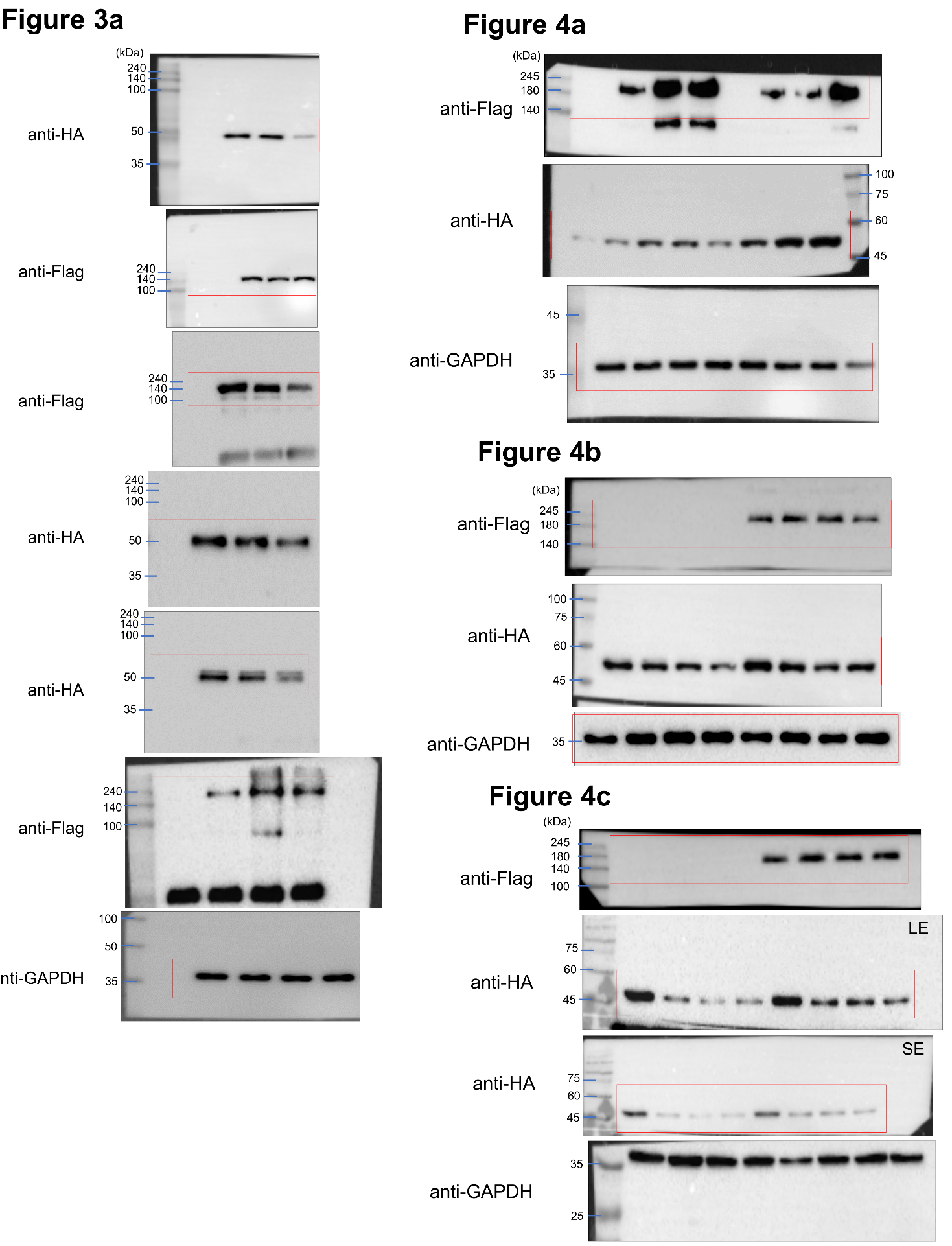

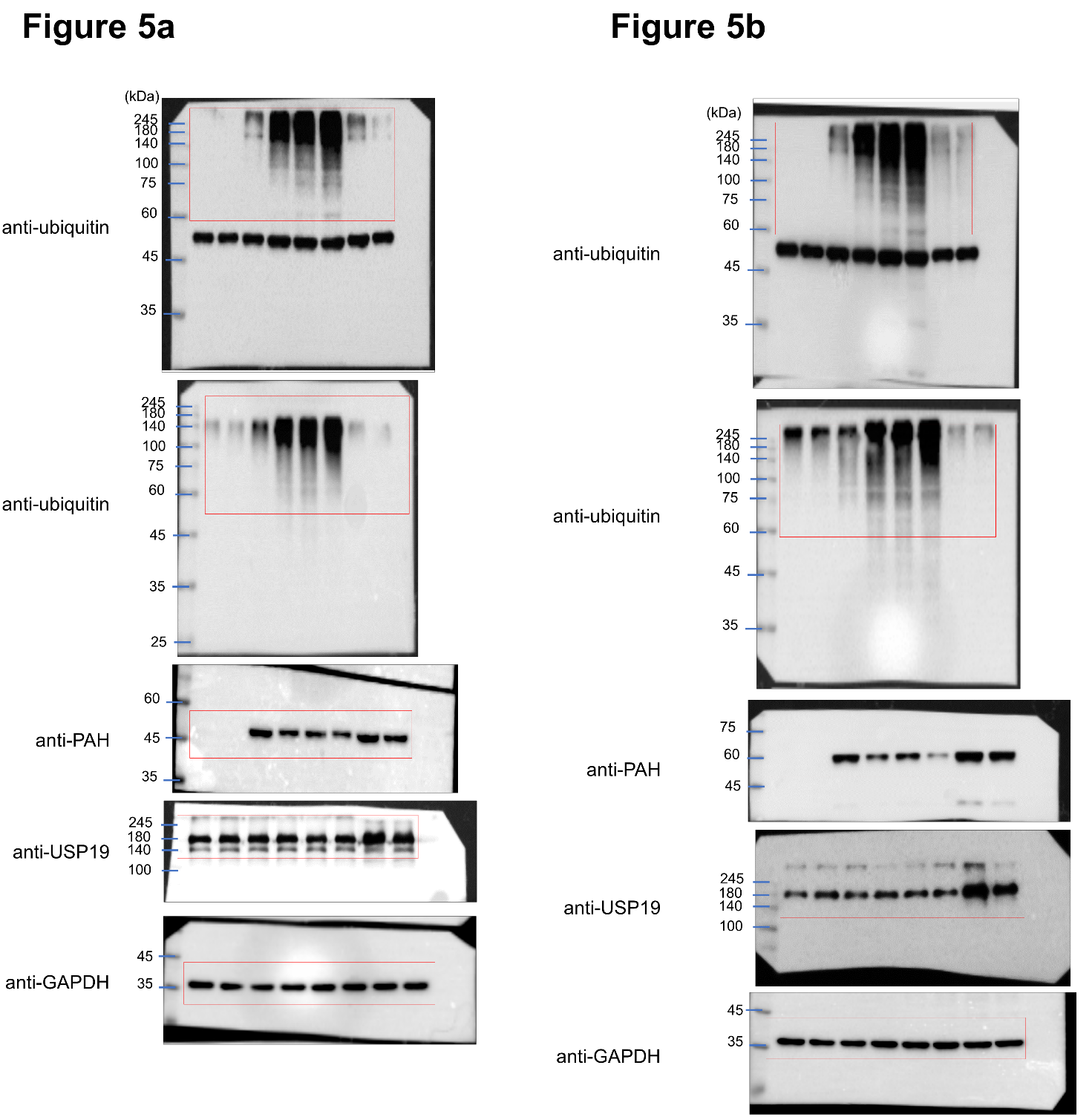
**

**
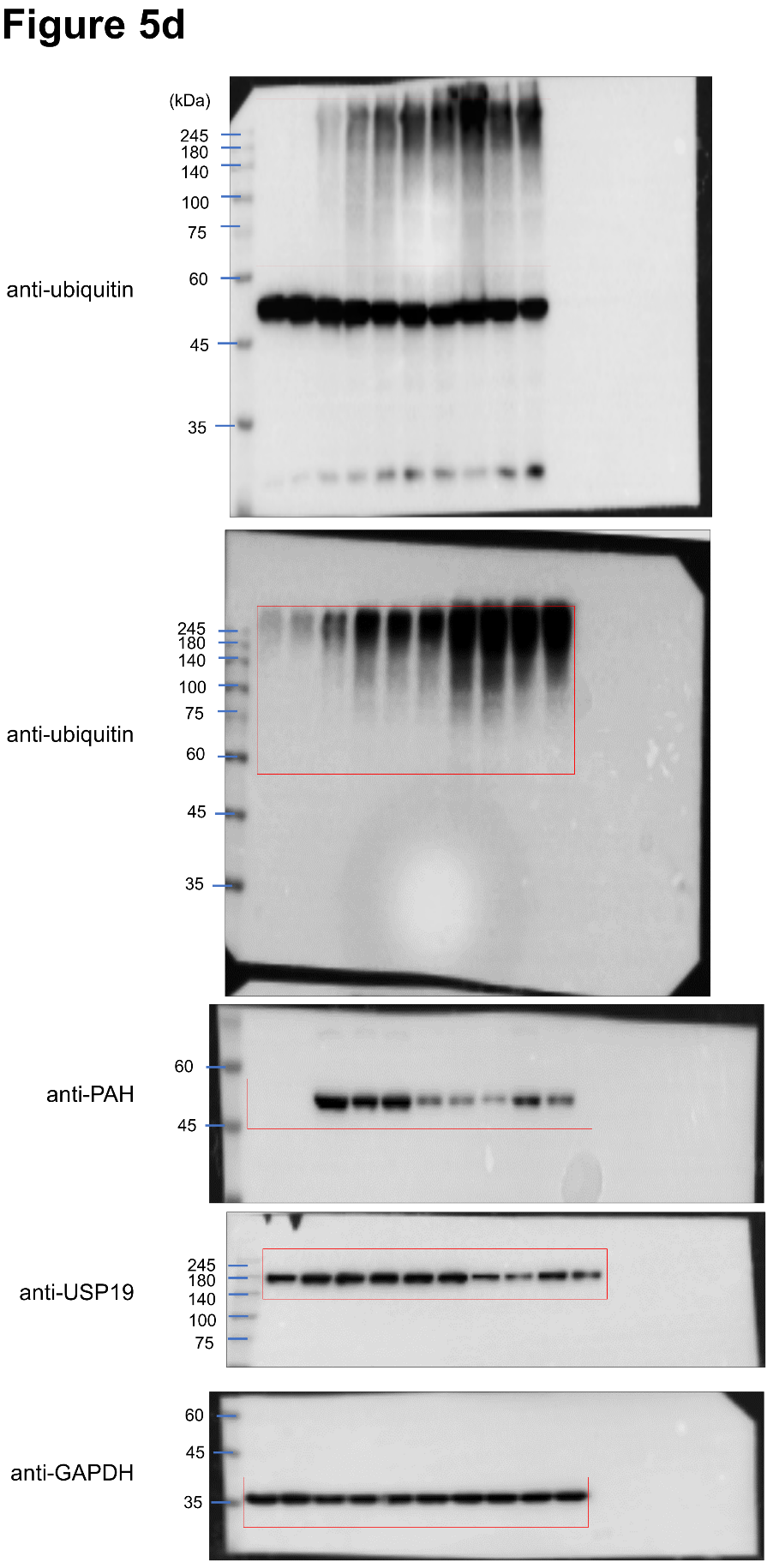

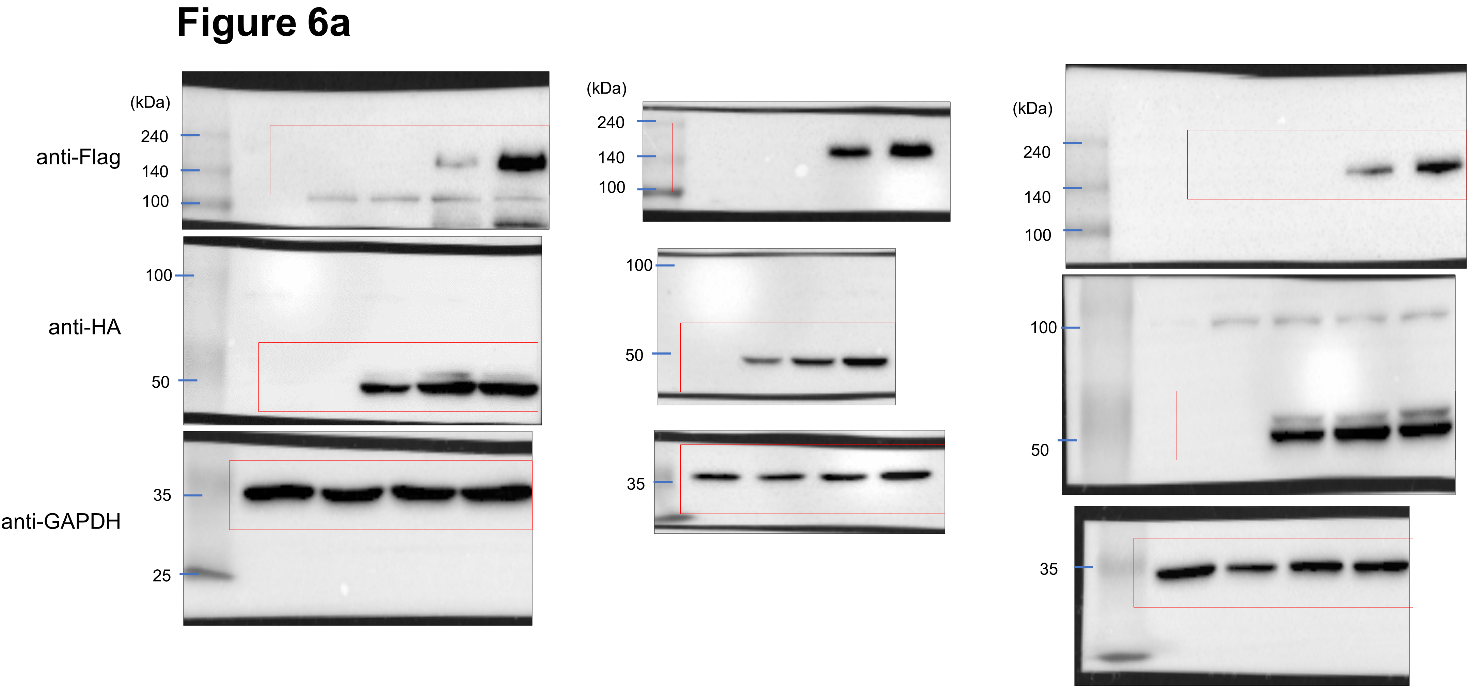
**

**
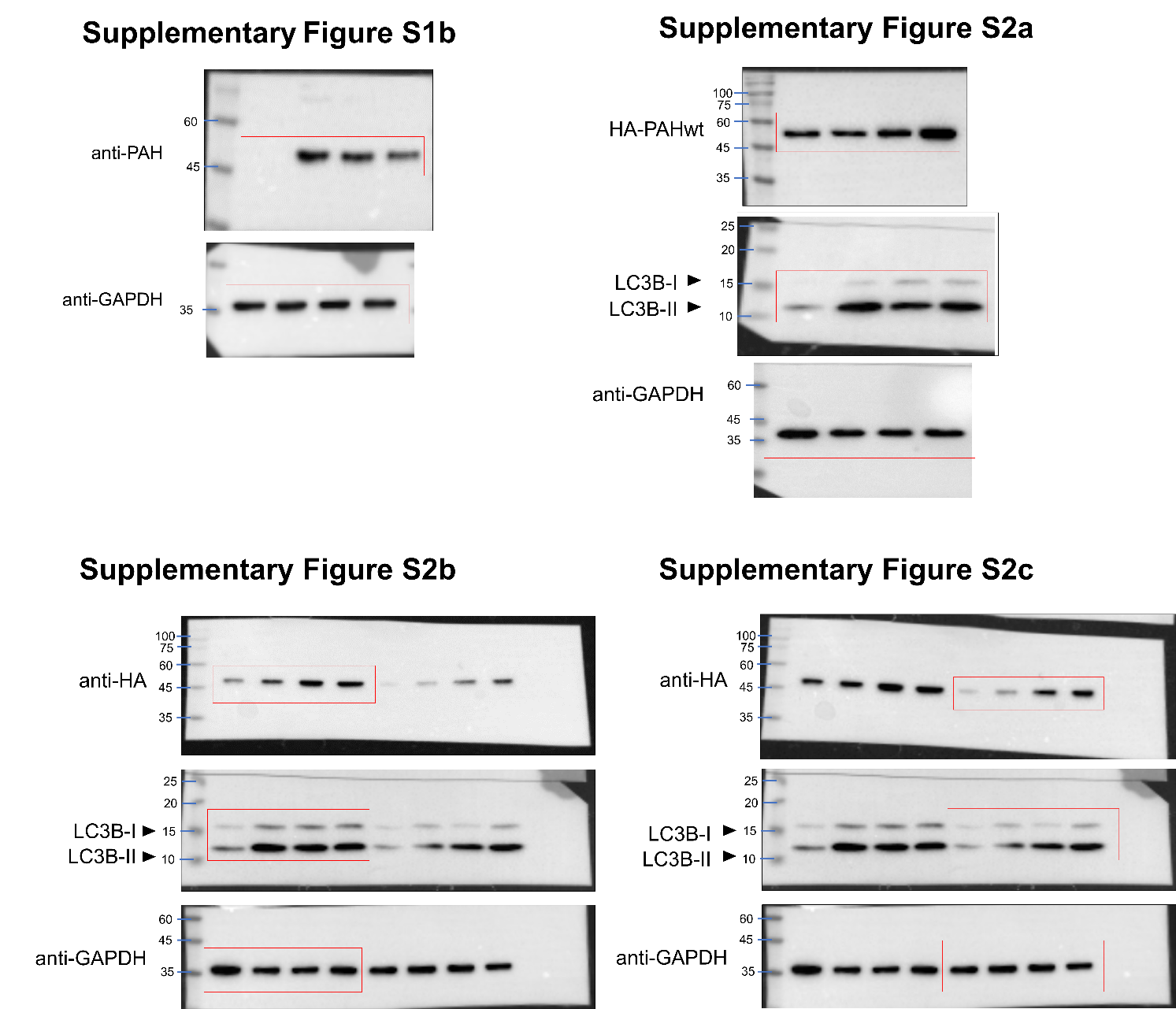
**

**
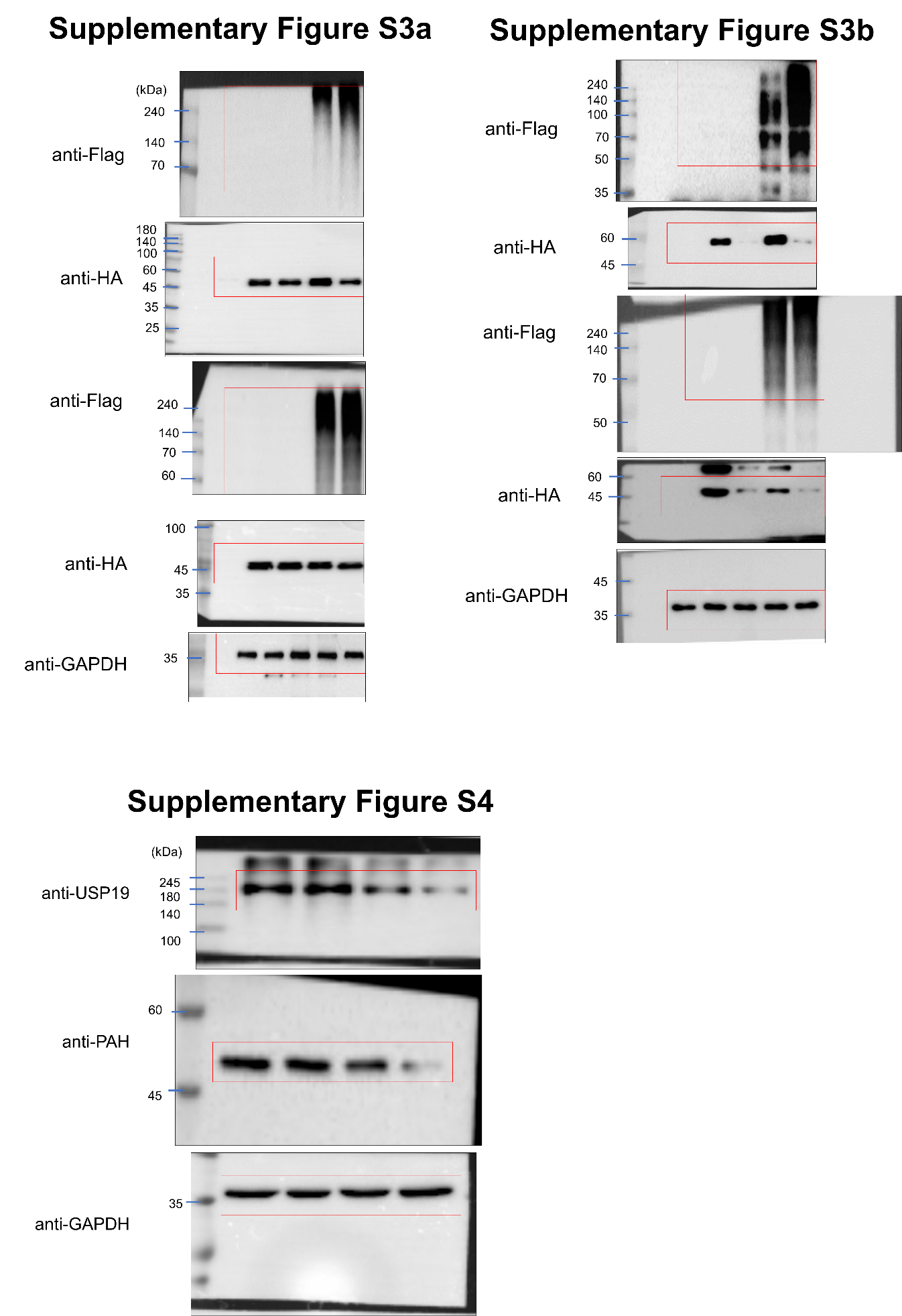
**

**Supplementary Table S1.** Oligonucleotides used for generating PAH variant by site directed mutagenesis.

| **Gene** | **PAH variant** | **Direction** | **Sequence (5’ to 3’)** |
| --- | --- | --- | --- |
| *PAH* | c.721C > T  (p.R241C) | FP | ACTGGTTTCTGCCTCCGACC |
|  |  | RP | GGTCGGAGGCAGAAACCAGT |
|  | c.728 G > A  (R243Q) | FP | TTTCCGCCTCCAACCTGTGGC |
|  |  | RP | GCCACAGGTTGGAGGCGGAAA |
